# Implementation of a trauma-informed, evidence-informed intervention for Latinx families experiencing interpersonal violence and child maltreatment: protocol for a pilot randomized control trial of SafeCare+®

**DOI:** 10.1186/s40814-020-00681-3

**Published:** 2020-10-08

**Authors:** Danielle L. Fettes, Gregory A. Aarons, Valerie Brew, Karla Ledesma, Jane Silovsky

**Affiliations:** 1 Department of Psychiatry, University of California, 9500 Gilman Drive, #0812, La Jolla, San Diego, CA 92093 USA; 2 Child and Adolescent Services Research Center, San Diego, CA USA; 3 South Bay Community Services, San Diego, CA USA; 4 Health Sciences Center, The University of Oklahoma, Oklahoma City, OK USA

**Keywords:** Domestic violence, Child welfare, Feasibility studies, Pilot projects, Early intervention, Home visitation

## Abstract

**Background:**

A consistently demonstrated overlap exists between the occurrence of domestic violence and child maltreatment, yet these issues are historically addressed by distinct systems and programming. The randomized control trial pilot study presented in this article adapts, implements, and tests a new approach for addressing family violence for Latinx families with co-occurring risk for domestic violence and child maltreatment. In doing so, this pilot study addresses the clear need for collaboration between the two fields and focuses on Latinx families, who often face specific challenges regarding seeking and receiving needed services. The primary aim of the current study is a pilot implementation of SafeCare+®, an evidence-based parenting curriculum (SafeCare®) augmented with a healthy relationships curriculum (SafeCare+®). The objectives are a reduction of family violence, improved communication, and a healthy home environment for children in Latinx families with co-occurring domestic violence and child maltreatment.

**Methods:**

This protocol outlines a feasibility, randomized control trial to examine the potential efficacy of SafeCare+. The pilot study is divided into two phases. Components of phase one involve developing a detailed implementation and evaluation plan, including a community needs assessment, determining screening and outcome measures, and assuring all components are culturally appropriate for the target population. Phase two implements the randomization of parents, who are involved in the child welfare system and referred for in-home parenting services, into SafeCare+ or SafeCare as usual. Participants complete assessments regarding mental health, provider-parent relationship, interpersonal violence experiences, and fidelity to the intervention. Analyses will focus on improvement on target outcomes for the intervention group, as well as comparison to the control group.

**Discussion:**

This study will provide evidence on the feasibility and potential effectiveness of an early intervention program aimed at improving communication skills and mental health and reducing incidents of violence for Latinx parents who are involved with the child welfare service system. The findings of the study will inform the decision to progress to a full scale, definitive randomized control trial to test the effectiveness of an intervention, delivered as part of home visitation, for improving outcomes for families with histories of domestic violence.

**Trial registration:**

ClinicalTrials.gov, NCT03041558; registered 2 February, 2017—retrospectively registered.

## Background

Child maltreatment and domestic violence (DV) are two vital public health concerns, often with considerable overlap and in extensive need of amelioration. Over 4 million cases of suspected child maltreatment were reported to state child protective service systems in the USA in 2017, with approximately 674,000 confirmed cases of maltreatment. The most common form of child maltreatment in the USA is neglect, accounting for over 74% of substantiated cases [1]. The individual and societal impacts of child maltreatment are well documented, as child trauma is associated with negative social, emotional, behavioral, and health effects on children and youth [2] at a notable economic cost [3–5]. The impacts of DV are similarly alarming. In the USA, over 1 in 3 (or 42.6 million) women experienced violence by an intimate partner over the course of a lifetime, with 6.6 million women reporting violence within the last year [6]. Approximately 15.5 million children are exposed to DV each year, with over 50% of DV incidents occurring in households with children [7].

### Co-occurrence of domestic violence and child maltreatment

Two decades of research have clearly documented a strong overlap between DV and child maltreatment, with national research indicating a 30 to 60% co-occurrence rate [8, 9]. These forms of family violence have a strong likelihood of co-occurring because they share several common risk factors, such as young maternal age, low level of education, and low socioeconomic status, among others [10, 11]. Children who have witnessed or experienced violence in their homes face an elevated risk of cognitive and emotional problems [12]. They often experience negative outcomes such as poor school performance, grade retention, juvenile delinquency, and teenage pregnancy [8]. Further, children who reside in homes with DV may show warning signs of stress, fear, and trauma [13].

While child maltreatment clearly has an adverse effect on healthy child development, the presence of DV can also interfere with parenting, placing children at additional risk. Children living in households with DV are more likely to experience emotional, physical, and sexual abuse [14]. Perpetrators often attempt to control their adult partners through methods such as intimidation, undermining their parental authority, or using children against the other parent [15]. Children may be injured when they attempt to intervene in a DV incident or get struck by objects intended to injure or frighten the parental victim. And, in the absence of physical abuse, DV may deteriorate the family environment and increase stress, impacting parents’ capacity to attend to their children’s needs.

Taken together, there is a clear need for collaborative efforts to provide appropriate services and to identify families struggling with relationship dynamics earlier in the cycle of family violence. Despite the growing awareness of the co-occurrence of DV and child maltreatment, however, programs and systems have historically responded separately. Existing parenting programs designed to address child maltreatment rarely include training in managing DV-related issues. However, the presence of DV has been shown to affect domains of improvement that are targeted by parenting programs. For example, a common element of many parenting programs is a focus on improving parent-child interactions. DV perpetrators often damage the parent-child relationship by victim-blaming and breeding distrust in what is typically the mother’s ability to protect her children [16]. Including content that addresses DV in the context of parenting programs creates a unique opportunity to both deliver services that acknowledge the complexity of the intersection of DV and parenting and provide early intervention to children and parents at risk for long term, negative outcomes.

### Need for culturally relevant and trauma-informed services for Latinx families

In addition to addressing service needs for families with co-occurring risk for DV and child maltreatment, the current study specifically focuses on Latinx families. The characteristics or consequences of DV among different cultures may vary. Attention to cultural differences among DV survivors can attune services to particular psychological, emotional, spiritual, economic, legal, and social needs. Cultural factors unique to the Latinx community that may impact the manifestation of DV include concepts such as *machismo* or *marianismo* [17]. These gender ideals encourage men to be dominant in intimate relationships and for women to demonstrate submissive and passive behaviors [18, 19]. Some evidence demonstrates that the effects of DV on Latina women lead to significantly greater trauma-related symptoms and depression as compared to non-Latina women [20].

The Latinx community in the USA covers a wide range of experiences, from recent immigrants with limited command of the English language, to families with generations of citizenship. Latina immigrants may have difficulty recognizing or responding to DV due to patriarchal values and beliefs in *familismo*, or family loyalty [21]. Even when DV is identified as an issue, Latina survivors can often face other obstacles in seeking help. Common barriers to help-seeking behaviors include lack of English proficiency, fear of deportation, and perception that law enforcement will not respond to domestic disputes [22]. Latinx families may face a range of additional risk factors such as recent immigration, resulting in great distance from traditional support systems, challenges related to cultural expectations, religion, and previous experiences of violence [23]. These factors may elevate traumatic stress reactions, indicating a clear need for collaborative efforts that focus on (1) identifying these families earlier in the cycle of family violence, (2) supporting them to garner the necessary skills to keep themselves and their families safe and healthy, and (3) providing safety planning and violence prevention.

### In-home parenting programs as a unique platform to provide early intervention for DV

The primary aim of the current study is a pilot implementation and testing of culturally relevant, trauma-informed programming to lead to a reduction of family violence, a resulting increase in family stability, improved communication, and a healthy home environment for children in Latinx families with co-occurring DV and child maltreatment. Specific objectives include the following:

1. Increased access to culturally specific, trauma-informed, evidence-informed interventions.
2. Increased understanding of the needs of the target population and how to meet them.
3. Improved collaboration, policies, and practices for effectively meeting the needs of the target population.
4. Reduced incidents of DV at case closure.
5. Improved mental health at case closure.
6. Improved communication skills at case closure.

The intervention being implemented is SafeCare+ (SC+), an evidence-based (EB) training curriculum for parents referred for child maltreatment, enhanced with the healthy relationships (HR) module. SafeCare (SC) has been shown to be widely accepted among the Latinx community [24]. The HR enhancement is a promising practice that may prevent and/or reduce family violence among Latinx families. The study will test the efficacy of the SC+ model, contributing to the DV field’s evidence base of effective practices that improve outcomes for Latinx families impacted by both DV and child maltreatment. The evaluation of the pilot implementation will impact and inform advocacy and practice change.

## Methods

### Setting

This randomized-control trial (RCT), pilot study was embedded within a large-scale, National Institute of Mental Health-funded study, the *Interagency Collaborative Teams to Scale-Up Evidence Based Practice* (ICT) [25]*.* The ICT study was a system-wide, mixed-methods implementation study focused on understanding inner and outer context factors associated with the effective implementation and sustainment of SC delivered to child welfare-involved parents, in a large, diverse child welfare service (CWS) system. The ICT study components (which include an assessment of the feasibility and acceptability of SC/SC+, the impact of service attrition on study outcomes, and fidelity to the intervention) serve as a foundation for the current pilot RCT.

The scale up of SC occurred across multiple community-based organizations (CBOs). These CBOs are contracted by CWS to deliver parent training, such as SC, via an in-home service delivery platform. Families involved with CWS are referred to the CBOs to receive SC because they had children between the ages of 0 and 11 and child neglect was the primary referral reason. As an EB curriculum, SC is structurally and behaviorally prescriptive on how its curriculum is to be delivered to the targeted parents by the home visitors. Most often, parents participate in the SC curriculum individually with the home visitor. Parent participant eligibility criteria for the ICT study were (1) at least 18 years of age; (2) were referred for child neglect; and (3) had at least one child in the family under age 12. Over 90% of eligible parents enrolled in the ICT study.

### Design

**Fig. 1:**
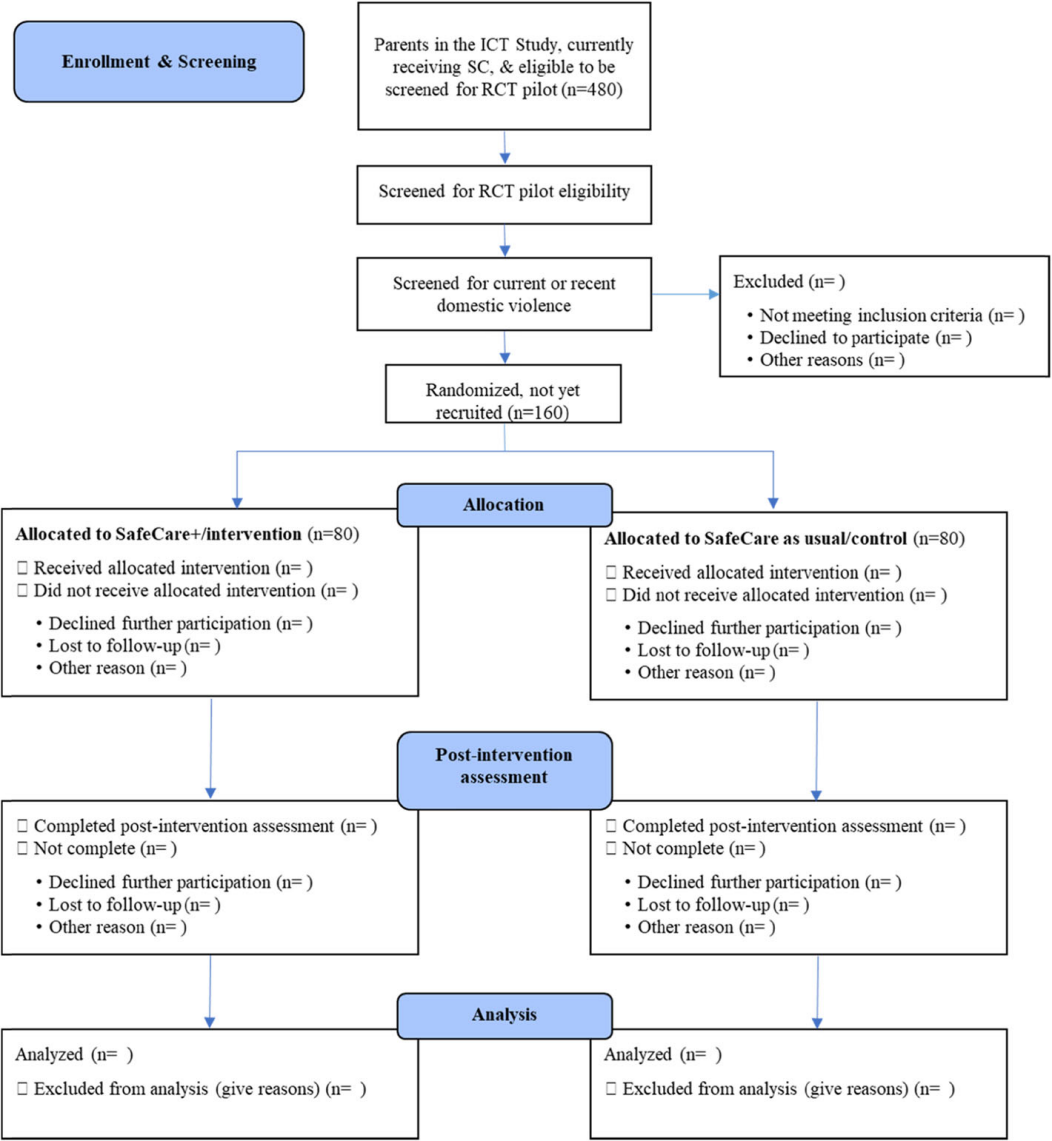
SafeCare+ study flow diagram

The pilot study is divided into two phases. During phase one, the first year of the study, community and academic partners collaborate to develop a detailed evaluation plan. The evaluation centers on the effective implementation of SC+ and measurement of relevant outcomes during phase two of the project—the RCT, pilot study. Components of phase one include (1) a community needs assessment, (2) refining the target population, (3) designing the evaluation plan, (4) determining screening and outcome measures, (5) conducting focus groups with key stakeholders, including DV providers and Latinx survivors, to assure that the chosen interventions and screening and assessment tools are culturally appropriate for the target population, and (6) ensuring the proper training of direct service staff. For phase two, the RCT pilot study will enroll at least 160 parents either into SC or SC+, allocating a minimum of 80 parents per condition. Parents will be randomized to SC or SC+ and within home visitor, such that every other eligible parent will be assigned to SC or SC+ within home visitor. The nested structure will be accounted for in multi-level analyses, see Fig. 1 (SafeCare+ study flow diagram).

### Eligibility criteria

Participants will be included in the RCT pilot study if the following criteria are met: (1) currently receiving SC services, meeting ICT inclusion criteria; (2) a primary caregiver identifying as Latinx; (3) over 18 years of age; and (4) at risk for DV. The criterion for risk of DV was intentionally set to be broadly inclusive of families who are struggling with psychological violence. Clients will be excluded from participation if there are immediate safety risks in the family.

#### Ethical considerations

Participants providing data in the study are service clients receiving family preservation/family reunification services (i.e., SC via home visitation) through partnering CBOs in the CWS system. These services are most often targeted at concerns over child neglect, and services are designed to strengthen the capacity of families to care for young children. Parental participation in the research study is voluntary, and parental data is maintained separate from CWS records. Parental declination to participate in the research study has no impact on service receipt—clients will receive CWS services independent of participation in the study.

### Interventions

#### Control group: SafeCare

SafeCare (SC) is an EB parenting curriculum, delivered via home visitation, for parents with preschool or school-age children who are at risk of or have been reported for child neglect, physical abuse, or both. SC targets improvement in skills related to home safety, health, and parent-child interaction [26]. These modules include a baseline assessment, training in knowledge and skills, and follow-up assessments to monitor change. Delivery of the program is based on the principles of behavioral analysis—service providers are trained to model skills, engage in ongoing measurement of observable behaviors, and to give constructive feedback during skills practice. SC program completion typically takes 18 to 20 home visiting sessions over 6 months. A number of studies attest to SC effectiveness and provide evidence on improved parental outcomes, including better management of their child’s health, increased home safety, and more positive and sensitive parent-child interaction [26–28]. In addition, SC has been demonstrated to be efficacious for reducing CWS recidivism relative to usual care [29]. Participants randomized to the control group will receive the EB, SC as usual.

#### Experimental group: SafeCare with Healthy Relationships (SafeCare+)

Participants randomized to the experimental group will receive SC+, an enhanced version of the SC program, which includes the healthy relationships (HR) curriculum, specifically designed for early intervention with family violence. The HR curriculum is intended to help parents recognize abusive patterns, develop healthier relationships, and improve communication skills with a range of adults, including intimate partners, family members, friends, and with their children. The HR module (1) provides information about how relationships can affect health and well-being for parent and child; (2) reviews qualities of healthy and unhealthy relationships; (3) assesses the type and quality of the parent’s important relationships; (4) empowers the parent to be an active participant in improving the quality of their personal relationships; and (5) teaches communication, problem-solving, and interpersonal skills to facilitate enhancing the quality of their personal relationships. The HR module is delivered in a similar skills-based format as other SC components, with ongoing measurement and feedback in areas of communication, problem solving, and interpersonal interactions.

### Feasibility outcomes

Acceptability of the intervention will be examined in both phases of the study. First, focus groups with the target population participants will determine the perceived fit of the intervention for the population. Focus groups will also provide information regarding the variety of experiences, barriers, and strengths that exist within various sub-groups of the target population (e.g., younger and older parents, recent immigrants). This information will be used to design and test various approaches to service delivery to meet the varied needs of the target population and to determine the feasibility for expanding the target population to include a prevention base for families at risk of DV. Second, satisfaction will be established by program participants on all intervention components. In addition to acceptability, SC+ limited effectiveness will be ascertained by comparisons between the experimental and control groups on key outcome measures, set to achieve at minimally medium effect size. Finally, practicality (i.e., perceived sustainability within the existing infrastructure) will be explored at the conclusion of the study via interviews with providers and service system leadership.

### Measures

All measures are client-reported and will be collected via a secure, web-based data entry system, on an internet-enabled tablet that service providers bring to each visit. At the end of each SC or SC+ visit, providers activate the participant survey by logging into the secure web-based data entry system on their assigned tablet computer and selecting the appropriate client identifier (a number randomly generated by the secure system). Once the survey has been activated, the service provider will give the client the tablet computer to complete the measures privately. In the infrequent circumstance that two parents are simultaneously participating in services, the assessments are completed by each, separately, while the other parent is engaged with the home visitor. All measures are available in both English and Spanish.

#### Screening measures

*Hurt*, *Insult*, *Threaten*, *Scream* (HITS) [30] is a 4-item instrument for DV screening. Items ask respondents how often their partner physically hurt, insulted, threatened with harm, and screamed at them. A score of 10.5 (range 4 to 20) was demonstrated to reliably differentiate victims of domestic abuse from family practice patients. The current pilot study includes parents who report a score of 7 or higher.

#### Outcome measures

*SC+ model fidelity* is assessed via client report, with clients completing a fidelity questionnaire at the end of every SC or SC+ session. The fidelity survey was adapted from the National SafeCare Training and Research Center training checklists, and the fidelity measure has been validated over the course of SC implementation [31]. Overall, monitoring of fidelity allows for measuring the effectiveness of the intervention as designed, as well as any practice changes which are naturally occurring and may need to be addressed.

*The Conflict Tactics Scales* (CTS2)*-short form* [32, 33] measures both the extent to which partners in a dating, cohabiting, or marital relationship engage in psychological and physical attacks on each other, as well as use of reasoning or negotiation to deal with conflicts. Participants are asked about the frequency of concrete acts and events within the past 6 months for their baseline CTS2 assessment, with the time frame changed to assessing if these acts and events occur within the past 2 months for the post-intervention assessment.

*The Center for Epidemiological Studies Depression Scale* (CES-D) [34] is a 20-item questionnaire, which assesses depressive symptomology. Designed as a short self-report tool, each item asks about the last week and is responded to using a 4-point format. The CES-D has high internal consistency and adequate test-retest repeatability.

*Relationship self-efficacy* (RSE) [35] is a 35-item measure evaluating relationship maintenance self-efficacy beliefs. Thirteen items relevant to the content of HR are included, across the three domains of emotional control, differentiation, and mutuality. Emotional control denotes the ability to appropriately regulate negative feelings with others. Differentiation is the capacity to assert clear interpersonal boundaries and express the need for separateness. Mutuality is defined as being able to provide care and receive support.

*Working Alliance Inventory* (WAI) [36] assesses working alliance between parents and their service providers. The current study employs the short-form, 12-item version of the WAI [37] and includes the components of goal, task, and bond. Goal is the extent to which the client and provider agree on goals of services. Task is the degree to which the client and provider agree on ways of achieving stated goals. Bond denotes mutual liking between client and provider.

*Relationship status* is a brief instrument created by the study team and intended to assess the respondent’s current intimate partner relationship or rationale for not currently engaging in one.

*Healthy relationships satisfaction* is a 14-item assessment of perspectives about the prescribed HR content, delivery of services, and service provider.

**Fig. 2:**
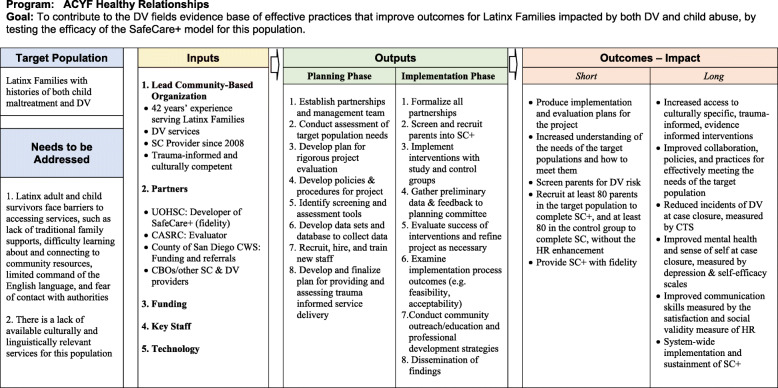
Logic model for the SafeCare+ pilot study

Figure 2 illustrates the framework of the research approach.

### Data collection points for outcome measures

**Fig. 3:**
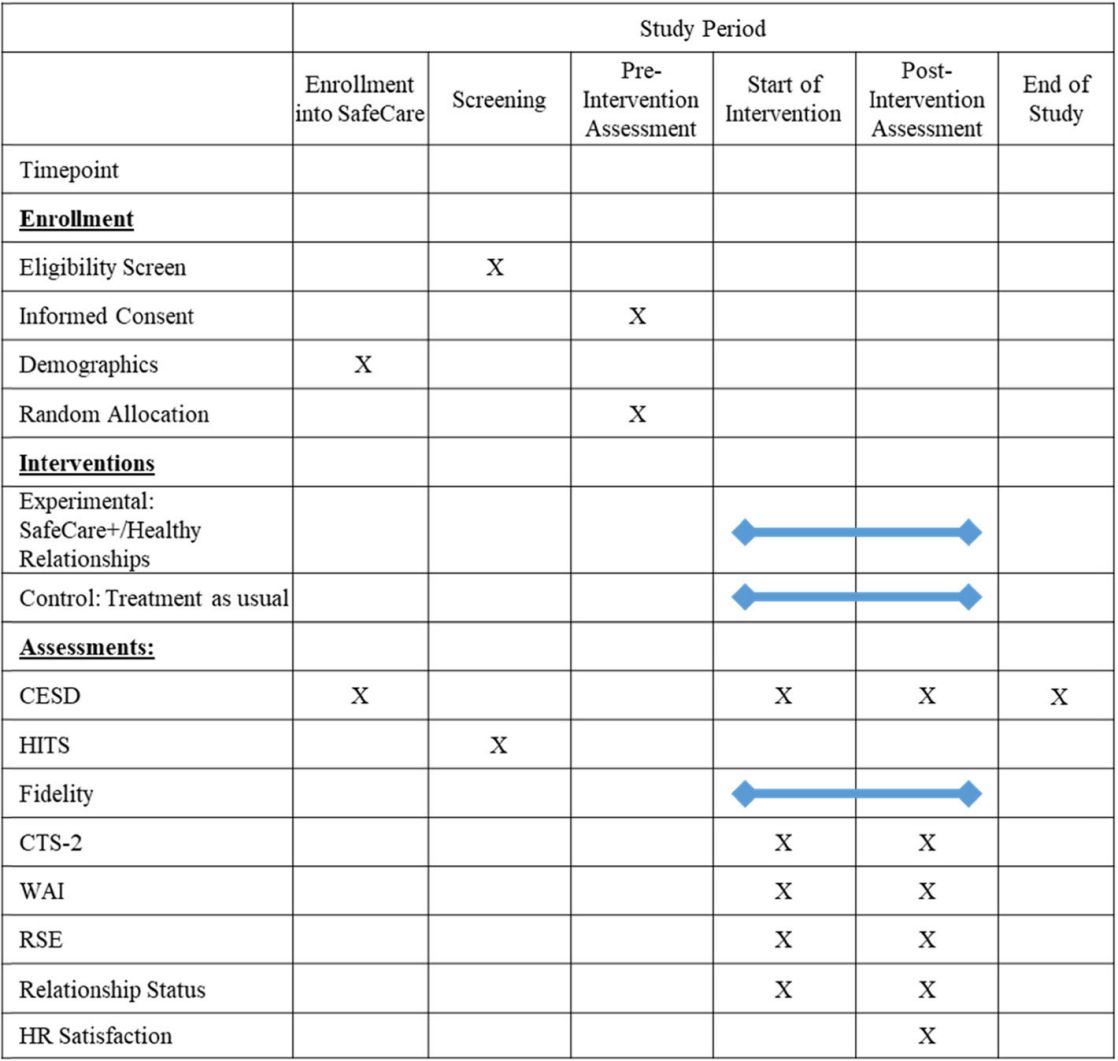
SPIRIT figure for SafeCare+ trial for data collection timings

Data collection for SC/SC+ fidelity will be conducted at each session of the intervention, throughout the course of service delivery. The CES-D is assessed at intake into SC, at the end of the intervention, and at the end of the study. The CTS-2, WAI, RSE, and relationship status measures are assessed at the beginning and conclusion of receiving the intervention (i.e., the HR curriculum, or the comparison time frame for the control condition). HR satisfaction is assessed at the conclusion of receiving the intervention, see Fig. 3 (SPIRIT figure for SafeCare+ trial for data collection timings).

### Data analyses

#### Quantitative analyses

Primary analyses will be based on generalized linear mixed models [38–40]. Mixed models are necessary for analyzing data from the proposed project because the data will have a three-level, hierarchical data structure in which measurements over time are nested within clients and clients are nested within provider. The models tested involve both fixed and time-varying covariates. All models will be multivariate in nature (i.e., testing all predictors simultaneously). Significance tests will focus on individual regression coefficients from the models. In addition, the final data set will offer rich opportunities for auxiliary or secondary analyses, including exploring the role of fidelity for mediating client outcomes. Dependent variables will be changed in (1) reports of DV on the CTS, (2) depression, (3) relationship self-efficacy, and (4) indicators of fidelity to SC and SC+.

#### Sample size

A conservative estimate for the duration of the proposed study is 160 parents, which provides an adequate sample size required to discern improvement, with a medium Cohen’s *d* effect size, on the proposed measures.

## Discussion

This pilot study targets families with co-occurring DV and child maltreatment and will demonstrate the impacts of an early intervention approach for Latinx parents with histories of DV. The study addresses the needs of this target population by providing SC, an EB home visiting program designed for families reported for, or at risk of, child maltreatment, supplemented through the addition of an HR curriculum that has been modified specifically for Latinx families (SC+). This study design is appropriate for the target population because it provides a culturally and linguistically refined approach that promotes the development of individual skills such as problem solving, active decision-making, conflict resolution, and healthy communication—both for enhanced parenting and within personal relationships. The HR module is intended to help parents learn about the characteristics of healthy and unhealthy relationships so they may begin to examine and improve the quality of their own relationships. It is anticipated that as parents become active agents in creating and maintaining healthy personal relationships, they will provide a safer and more emotionally healthy environment for their children and will enhance their parenting effectiveness. Thus, the goals are to improve family stability by reducing conflict, improve communication in all areas of the parents’ life, and provide positive modeling for children. All services will promote integrity and self-sufficiency, improve access to resources, and increase the safety of adult and child survivors of DV and child maltreatment.

A primary aim of the RCT, pilot study is to demonstrate the effectiveness of the HR curriculum. A significant advantage of the design of the pilot study is the embedded nature of the RCT into a larger study. Substantial infrastructure support with regard to recruitment and data collection serve as an important foundation to success. A primary challenge of the RCT pilot study is that the inclusion of the HR curriculum into the existing service delivery system extends the length of services by 30% (~ 2 months). As a result, parents assigned to the intervention condition may not actually receive the entirety, or in some cases any, of the intervention due to the service requiring more time than the CWS timeline for length of service delivery. A study limitation is that parents with literacy challenges may have difficulty completing the outcome measures to determine effectiveness. In addition, the cultural appropriateness of outcome measures is a primary consideration. The first phase of the study is intended specifically to collaborate with technical assistance providers, community partners, and Latinx parents to ensure that the data collection efforts are aligned with the trauma-informed approach of the study.

Results from this study will inform the ways in which the CWS system of care may be strengthened to sustain long-term safety and well-being of children, while also providing efficacy data for a large, system-wide randomized control trial of the intervention. In addition, service providers are gaining multiple experiences with the provision of trauma-informed practice and intentional approaches to working with children and families with histories of DV. This pilot study provides an important foundational step to determining how DV services may be integrated into CWS home visitation programs, setting a platform for continued work with this population.

## Data Availability

The datasets generated during and/or analyzed during the current study are not publicly available due to ongoing recruitment and the sensitive nature of the study population, but are available from the corresponding author on reasonable request.

## Abbreviations

CBO: Community-based organization
CWS: Child welfare services
DV: Domestic violence
HR: Healthy relationships
RCT: Randomized control trial
SC: SafeCare
SC+: SafeCare with Healthy Relationships
